# Harnessing Available Evidence in Single-Case Experimental Studies: The Use of Multilevel Meta-Analysis

**DOI:** 10.5334/pb.1307

**Published:** 2024-10-25

**Authors:** Wim Van den Noortgate, Patrick Onghena

**Affiliations:** 1 Methodology of Educational Sciences Research Group, Faculty of Psychology and Educational Sciences, KU Leuven, Belgium; 2 Itec, an imec research group at KU Leuven, Belgium

**Keywords:** single-case experimental design, SCED, single-subject experimental design, meta-analysis, multilevel

## Abstract

The use of multilevel models to combine and compare the results of multiple single-case experimental design (SCED) studies has been proposed about two decades ago. Since then, the number of multilevel meta-analyses of SCED studies steadily increased, together with the complexity of multilevel models used. At the same time, many studies were done to empirically evaluate the approach in a variety of situations, and to study how the flexibility of multilevel models can be employed to account for many complexities that often are encountered in SCED research, such as autocorrelation, linear and nonlinear time trends, specific designs, external event effects, multiple outcomes, and heterogeneity. In this paper, we give a state-of-the-art of the multilevel approach, by making an overview of basic and more extended models, summarizing simulation results, and discussing some remaining issues.

A single-case experimental design (SCED) is a design in which a single unit or case (often a human participant) is measured repeatedly under multiple conditions that are manipulated by the experimenter (Onghena, 2005). By comparing the responses across time and across conditions on one or more outcome variables, one can get an insight in the effect of the conditions for the case that is studied. SCED studies are increasingly used to assess the effectiveness of interventions (Kratochwill & Levin, 2010). A first reason is that it has become clear that the use of SCEDs has the potential to yield internally valid information (Shadish et al., 2002). This comes along with an increased quality of SCED studies, partly due to the availability of clear guidelines on the setup and analysis of SCEDs (e.g., Kratochwill et al. 2010). For instance, un-replicated nonrandomized AB-designs with a fixed baseline phase (phase A) followed by a treatment phase (phase B) are nowadays hardly used, because of the limited internal validity: a change in behavior after the introduction of a treatment cannot be confidently attributed to a treatment effect, as it can be due to the effect of a hidden or unhidden external factor (such as an event occurring around the start time of the intervention). An external factor is a less plausible explanation of the association between an outcome and the condition, and hence the internal validity is higher, if the start of the intervention was randomly assigned to a time point, when the outcome returns to the baseline level when the treatment is withdrawn in a third phase (in a so-called ABA design), or when the effect is observed in several settings or for several cases. A second reason is that SCEDs have clear advantages, especially compared to group comparison experimental design (GCED) studies: Because in SCED studies, the effect of individual cases is evaluated rather than the average effect for a population of cases, they are applicable even if only one or a few cases are available or of primary interest to the researcher, they can be used to evaluate a treatment that is tailored to the case at hand, and they are relatively cheap. Therefore, it comes as no surprise that SCEDs are often used in fields like (special) education (Kratochwill & Levin, 2014; Riley-Tillman & Burns, 2009; Shadish & Rindskopf, 2007), psychology (Shadish, 2014; Smith, 2012), and health sciences (Janosky et al., 2009).

Although a SCED study may be primarily designed to assess the effectiveness of an intervention for one specific case, many studies replicate the design over multiple cases, in order to investigate the generalizability of the treatment effect. One of the crucial criteria defined by the What Works Clearinghouse (2020) to rate SCED studies’ value is the inclusion of at least three attempts at three different points in time to demonstrate a treatment effect. This can for instance be achieved in the popular multiple baseline design (MBD) across participants, in which each of a few participants is measured a couple of times in a baseline phase, followed by measurements during or after the introduction of a treatment, while the start point of the intervention is varied over participants. Beside the replication within studies, SCEDs are also often replicated over studies. One can distinguish between direct and systematic replication (Kazdin, 2010). In a direct replication, the treatment is applied to new cases under (almost) the same conditions, allowing researchers to get a more precise insight in its effect. In a systematic replication, features of the treatment are systematically manipulated, allowing researchers to extend their insight in what works and under what conditions. Over the years, many treatments were evaluated many times by SCEDs, resulting in a rich body of research evidence. A meta-analysis of this evidence has the potential to reach more precise estimates of the general effects of interventions, to shed light on the variation between participants in the size of the effect, and importantly, to explore what characteristics of participants, treatment implementation, or context moderate the effect of the intervention. Therefore, a meta-analysis can yield a better theoretical understanding of the treatment and can be of immediate practical relevance.

The combination of data from multiple studies however requires reliable research methods: It is important that the data are collected in a systematic way in order to avoid bias, that they are critically appraised because the quality of the data immediately affects the quality of the conclusion, that the data are aggregated in a systematic way, and that the research report is transparent and accurate. SCED research has a strong tradition of drawing conclusions based on visual inspection of time series graphs (Manolov & Moeyaert, 2017), especially looking at six features (see e.g., What Works Clearinghouse, 2020): within phases, researchers look at the mean level, the time trend and the variability of the outcome around a straight line; across phases, researchers look at the immediacy of the effect, overlap in scores between adjacent phases and the consistency of data in similar phases (e.g., in two baseline phases). A major strength of visual analyses is that they can account for the richness of the data and therefore can help in drawing causal conclusions, whereas statistical models might oversimplify and therefore miss important patterns in the data such as external event effects. Although the use of trained researchers and clear protocols can lead to consistent interpretations of SCED data (Ferron and Jones, 2006; Kahng et al. 2010; Kratochwill et al., 2011; Lane & Gast, 2014; Manolov & Vannest, 2023), there are concerns about both the Type I and Type II error rate when using visual inspection to decide on the existence of a treatment effect (Allison et al., 1992; Harrington & Velicer, 2015). Especially when drawing conclusions based on a set of SCED studies, we therefore believe in line with for instance Manolov et al. (2014), that it is important to supplement visual techniques with statistical techniques.

Although the utility of statistical meta-analyses of SCED research has initially stirred up some debate (see e.g., Strain et al., 1998), gradually the focus of the discussion moved towards the question how a meta-analysis of SCED studies can be done in order to yield valid results that account for the idiosyncrasies in the data (Burns, 2012; Horner & Kratochwill, 2012; Schlosser & Sigafoos, 2008).

At the same time, meta-analyses are increasingly used to aggregate SCED data over studies. In their systematic review of SCED meta-analyses between 1985 and 2015, Jamshidi et al. (2023) found 178 published and unpublished meta-analyses, 94 (or 53%) of them from the last five years of this time span. They also found that in these meta-analyses, the majority used the ‘percentage of non-overlapping data’ (PND), this is the percentage of datapoints under the treatment condition that exceeds the most extreme datapoint under the baseline condition (Mastropieri & Scruggs, 1985), as an effect size measure. These effect sizes are often summarized across cases and studies by calculating an average, a median and/or a range. Unfortunately, the PND suffers from sensitivity to outliers and ceiling effects, and does not account for possible time trends (Carter, 2013; Parker & Vannest, 2009; Wolery, 2010). Moreover, its sampling variance is not known, which is valuable in meta-analysis for two reasons. First, weighting effect sizes by the inverse of their variance when calculating an average effect size results in the most precise overall effect size estimate. Second, if we know the sampling variance of the individual effect sizes, we can also obtain the standard errors of the overall effect size estimates that can be used to calculate confidence intervals and perform significance tests. Although the search to overcome these limitations has led to various alternative nonoverlap measures (Parker et al., 2011), we advocate an alternative approach to aggregate data from multiple SCED studies: the use of multilevel models (which is more or less a synonym for hierarchical linear models or mixed models) (Nugent, 1996; Rindskopf & Ferron, 2014; Shadish et al., 2013; Van den Noortgate & Onghena, 2003a, 2003b, 2007, 2008). The multilevel modelling approach indeed allows answering the meta-analytic questions discussed above and avoids the drawbacks of simpler approaches like averaging PND statistics. Since the use of multilevel models for aggregating SCED data has been proposed about two decades ago, many studies evaluated and further extended this approach. Yet, Jamshidi et al. (2023) found that only 22 out of the 178 meta-analyses used a multilevel approach, with 19 (86%) of them being published in the last five years of the studied period. It looks like the methodological advancements are only gradually entering the practice of SCED meta-analysis. Possible explanations are that the approach is still not well-known and that descriptions of models and techniques are scattered over the literature. The purpose of this paper is therefore to give a state-of-the-art of the methodological research on the multilevel approach to SCED meta-analysis, in order to help applied researchers to understand the principles and the opportunities of multilevel SCED meta-analyses, so that they can choose a model that fits the specificities of their data and research questions. In the next sections, we will first introduce the basic models to aggregate raw SCED data or SCED effect sizes and discuss some studies evaluating their performance. Afterwards, we will present some extensions of the models used to aggregate raw data or effect sizes. Next, we discuss the estimation of the model parameters and we end with a discussion of some remaining challenges.

## Basic Multilevel Models for the Meta-Analysis of SCED Data

### The multilevel analysis of raw sced data

In a SCED study, we have repeated measures per case. Thanks to the tradition within SCED research to report time series graphs for visual inspection, raw data can often be retrieved using data extraction software (Moeyaert, Maggin et al., 2016), and be statistically (re-)analyzed afterwards. A simple regression model that could be used to analyze data from one case measured under a baseline and a treatment condition is a model regressing the outcome variable *Y* on a treatment dummy indicator variable (*D*) that is equal to 1 if the observation was made during the treatment condition, 0 otherwise (Equation 1).


1 \[{Y_i}\,{\mathrm{ = }}\,{\beta _0}\,{\mathrm{ + }}\,{\beta _1}{D_i}\,{\mathrm{ + }}\,{e_i}\]

The residuals are assumed to follow a normal distribution with zero mean and an unknown standard deviation. The intercept, *β*_0_, refers to the expected value under the baseline condition. The regression weight of the treatment dummy variable, *β*_1_, refers to the change in the expected value when the case was observed under the treatment condition rather than under the baseline condition. This difference in means therefore can be considered as a treatment effect measure. Note that testing this coefficient is equivalent to using a two-sample *t*-test, assuming a common population variance.

Often a few cases are included in a study, for instance when a replicated AB-phase design or an MBD across participants is used. Jamshidi et al. (2023) found that the average number of cases per primary study in the SCED meta-analyses was 4. In this situation the SCED data have a two-level structure: repeated measurements (Level 1) are nested in cases (Level 2). If we have data from multiple studies, cases are in turn nested within studies (Level 3). Equation 1 can be extended to a two-level model to aggregate the data across cases, or to a three-level model to aggregate the data across studies (Van den Noortgate & Onghena, 2003a, 2007, 2008). The three-level model reads as:


2 \[
{Y_{ijk}} = (\beta_{0} + v_{0k} + u_{0jk}) + (\beta_{1} + v_{1k} + u_{1jk})D_{ijk} + e_{ijk}
\]

Note that we now use three indices: *i* to refer to the measurement occasion, *j* to refer to the case, *k* to refer to the study. The model includes four additional residuals, also called random effects: *v*_0*k*_ and *v*_1*k*_ are random study effects that express how the baseline level and treatment effect for study *k* deviate from the overall baseline level and treatment effect (*β*_0_ and *β*_1_), and *u*_0*jk*_ and *u*_1*jk*_ express how case *j* from study *k* deviates from the mean baseline level and treatment effect from study *k*. Typically it is assumed that the residuals at each level (the *v*’s at the study level, the *u*’s at the case level and the *e*’s at the measurement level) are identically and independently multivariate normally distributed with zero mean and unknown variance-covariance matrix. Parameters that are estimated are the (mean) regression coefficients, the *β*’s, also called fixed coefficients, and the (co)variance parameters of the random effects. Sometimes the covariances between random effects at a given level are constrained to zero in order to reduce the number of parameters to be estimated (especially when the number of units at this level is limited). The parameters are often estimated and tested using (restricted) maximum likelihood, (RE)ML, as implemented in software for multilevel or mixed models, such as SAS PROC MIXED, the Mixed procedure of SPSS or the *lme4* package of R. Whereas the fixed parameters from Equation 2 give an idea of the average baseline level and treatment effect across cases, the variance parameter at the first level refers to the stability of the scores within cases and the variances at the second and third level to the amount of variability of the baseline and treatment effect over cases and over studies. The covariance at the second and third level indicates whether the baseline level and treatment effect are correlated over cases and over studies, respectively. One could also get improved estimates of the case- and/or study-specific effects by using empirical Bayes estimates. These empirical Bayes estimates are more precise than the estimates one would obtain by using the data of the case or study only, because they ‘borrow strength’ from other cases or studies (Ferron, Farmer, & Owens, 2010; Van den Noortgate and Onghena, 2003a).

An advantage of the multilevel modelling framework is its amazing flexibility, allowing researchers to adapt the model to the type of data and the research questions of interest. To give an example, suppose the performance of cases is measured during a baseline phase followed by a treatment phase and suppose the outcome variable shows a positive linear trend over time. In this scenario, the use of Equation 2 is expected to result in an overestimation of the treatment effect, because also the time trend contributes to an increased expected performance in the second phase. This overestimation may easily be avoided by including a time-related variable (e.g., the observation number) as a predictor. For instance, one could use a multilevel extension of the model proposed by Center, Skiba, and Casey (1986) that regresses the outcome on the time trend, the treatment indicator, and the interaction between both:


3 \[\begin{array}{ccccc}
{Y_{ijk}}\,{\mathrm{ = }}\, & \left({{\beta _0}\,{\mathrm{ + }}\,{v_{0k}}\,{\mathrm{ + }}\,{u_{0jk}}} \right)\,{\mathrm{ + }}\,\left({{\beta _1}\,{\mathrm{ + }}\,{v_{1k}}\,{\mathrm{ + }}\,{u_{1jk}}} \right)Tim{e_{ijk}}\,{\mathrm{ + }}\,\left({{\beta _2}\,{\mathrm{ + }}\,{v_{2k}}\,{\mathrm{ + }}\,{u_{2jk}}} \right){D_{ijk}}\\
& {\mathrm{ + }}\,({\beta _3}\,{\mathrm{ + }}\,{v_{3k}}\,{\mathrm{ + }}\,{u_{3jk}})Tim{e_{ijk}}\,{\mathrm{*}}\,{D_{ijk}}\,{\mathrm{ + }}\,{e_{ijk}}
\end{array}\]

By centering the time variable around the start time of the intervention, the fixed effects (*β*_0_, *β*_1_, *β*_2_, and *β*_3_) can be interpreted as the expected value at the end of the baseline phase, the linear time trend during the baseline phase, the immediate effect of the treatment, and the change in time trend when going from baseline phase to treatment phase. We therefore have now two coefficients (*β*_2_, and *β*_3_) that refer to treatment effects.

A second extension is the inclusion of characteristics of cases and/or studies as predictors, in an attempt to explain the variance found between cases and/or studies. For instance, Equation 4 includes one case characteristic (*Age*) representing the chronological age of the case as well as one study characteristic (*App*) indicating whether or not the treatment in the study was supported by means of a digital app. Whereas *β*_1_ and *β*_2_ now can be understood as the effect these characteristics have on the expected baseline level, *β*_4_ and *β*_5_ refer to the moderating effect these characteristics have on the treatment effect. Additional case and study characteristics can be included, and the effect of a case-characteristic on the baseline and treatment effect can be allowed to vary over studies, by including additional random effects.


4 \[\begin{array}{ccccc}
{Y_{ijk}}\,{\mathrm{ = }}\, & ({\beta _0}\,{\mathrm{ + }}\,{\beta _1}Ag{e_{jk}}\,{\mathrm{ + }}\,{\beta _2}Ap{p_k}\,{\mathrm{ + }}\,{v_{0k}}\,{\mathrm{ + }}\,{u_{0jk}})\\
& {\mathrm{ + }}\,({\beta _3}\,{\mathrm{ + }}\,{\beta _4}Ag{e_{jk}}\,{\mathrm{ + }}\,{\beta _5}Ap{p_k}\,{\mathrm{ + }}\,{v_{1k}}\,{\mathrm{ + }}\,{u_{1jk}}){D_{ijk}}\,{\mathrm{ + }}\,{e_{ijk}}
\end{array}\]

Multilevel analyses using Equations 2 to 4 assume that across studies, the outcome variable is measured on the same scale. Often this is not the case. For instance, in one study, the amount of problem behavior may be counted in intervals of 10 minutes, in another study, the amount may be counted in intervals of one hour. Other factors held constant, the expected scores for the second study therefore are six times larger. As a result, also the residual standard deviation (*σ*_*e*_) will be six times larger. Therefore, Van den Noortgate and Onghena (2003b, 2008) proposed to standardize the data when different scales are used, by performing an ordinary least squares (OLS) regression analysis per case to estimate the residual standard deviation and divide the outcome scores by this estimated standard deviation, before combining the data using a multilevel model.

### The multilevel analysis of effect size data

An alternative approach to combine the results of multiple SCED studies is to calculate effect sizes in a first step, and then combine these effect sizes using meta-analytic methods in a second step. As noted earlier, *β*_1_ from Equation 1 is the difference between the expected score under the treatment and the baseline condition, and this mean difference can be used as an effect size measure. If we would standardize the data as described above, that is, by dividing the data by the residual standard deviation estimate, *β*_1_ becomes a standardized mean difference (SMD), very similar to the SMD proposed by Cohen (1969) but this time expressing the effect for a single case, rather than the overall effect across study participants. In a first step, we estimate these case-specific effect sizes, either by performing OLS regression analyses for each case separately, or by deriving the measure from reported summary statistics or test statistics. We will refer to the OLS estimate of *β*_1_ as *b*_1_. The sampling variance can be estimated by using the squared standard error estimates from the OLS regression. However, because these do not account for the additional variation due to using an estimate of the residual standard deviation to standardize the data, it is slightly better to use the formula derived for the sampling variance of GCED SMDs (Equation 5; Hedges, 1981), but this time *n*_*C*_ and *n*_*E*_ refer to the number of measurements under the baseline and treatment condition, respectively, rather than to the number of study participants in the control and treatment group.


5 \[\sigma _{{b_1}}^2\, \cong \,\frac{{{n_E}\,{\mathrm{ + }}\,{n_C}}}{{{n_E}{n_C}}}\,{\mathrm{ + }}\,\frac{{b_1^2}}{{2({n_E}\,{\mathrm{ + }}\,{n_C})}}\]

For the second step, one can again use a multilevel model to combine the effect sizes:


6 \[{b_{1jk}}\,{\mathrm{ = }}\,({\beta _1}\,{\mathrm{ + }}\,{v_{1k}}\,{\mathrm{ + }}\,{u_{1jk}})\,{\mathrm{ + }}\,{r_{1jk}}\]

As in a typical meta-analysis, also in this (three-level) meta-analysis, the variance of *r*_*1jk*_ can be estimated prior to performing the meta-analysis (using Equation 5), and therefore can be considered as known in the meta-analysis. Multilevel or meta-analysis software, including SAS PROC MIXED or the *metafor* package for R (Viechtbauer, 2010) can be used to fit this model, as long as it allows to include two random effects and to constrain the level-1 variance to the previously estimated values. The interpretation of the model parameters, *β*_1_, \[\sigma _{{v_1}}^2\], and \[\sigma _{{u_1}}^2\], is exactly the same as the interpretation of *β*_1_, \[\sigma _{{v_1}}^2\], and \[\sigma _{{u_1}}^2\] from the raw data model (Equation 2), namely, the mean treatment effect, the between-study variance and the between-case variance in the treatment effect. A major difference however is that when using (standardized) mean differences, information on the baseline level is ignored and cannot be used to estimate the mean baseline level and variation in the baseline level (*β*_0_, \[\sigma _{{v_0}}^2\], and \[\sigma _{{u_0}}^2\] from Equation 2). But when the interest is only in the treatment effects, both approaches are theoretically equivalent.

A drawback of standardizing (raw or effect size) data per case is that the resulting effect sizes are not immediately comparable to Cohen’s *d*, the commonly used SMD metric for GCED studies. Whereas we proposed to standardize the outcome variable or the regression coefficient by dividing them by the residual within-case standard deviation, Cohen’s *d* is the ratio of the mean difference over the between-subject standard deviation within conditions. Note that scores of persons are also influenced by intra-individual variability. Assuming that the effect in a SCED and GCED study is the same, the relation between both effect sizes is therefore (Van den Noortgate & Onghena, 2008):


7 \[\frac{{SM{D_{GCED}}}}{{SM{D_{SCED}}}}\,{\mathrm{ = }}\,\sqrt {\frac{{\sigma _e^2}}{{\sigma _e^2\,{\mathrm{ + }}\,\sigma _u^2}}} \]

This means that there is a risk of misinterpreting the size of the SCED effect, as commonly rules of thumb for the interpretation of the size of SMDs (e.g., Cohen, 1969) apply to GCED effect sizes only. Moreover, it means that GCED and SCED effect sizes cannot directly be combined and compared in a meta-analysis. In order to make the effects sizes for individual studies or across studies comparable with those of GCED, Van den Noortgate and Onghena (2008) proposed to perform a multilevel analysis of the raw SCED data to estimate the within and between-case variances, and use these estimates to convert the SCED SMDs. They also proposed a model to combine effect sizes from both types of designs in one meta-analysis, accounting for the fact that a SCED typically reports one effect size per case, whereas a GCED study typically reports one effect size over all cases. An alternative approach is the use of the so called Between-Case SMD (BC-SMD) measure proposed by Hedges and colleagues (Hedges et al., 2012, 2013; Pustejovsky et al., 2014; Shadish et al., 2014) that immediately uses the between-case variability to standardize the mean difference per study, and corrects for small sample bias. In the same vein, Joo et al. (2022) proposed to standardize the raw or aggregated data per study by dividing them by \[\sqrt {\hat \sigma _e^2\,{\mathrm{ + }}\,\hat \sigma _u^2}\].

A drawback of the approach proposed by Van den Noortgate and Onghena (2008) is that the conversion factor in Equation 7 has to be estimated and the uncertainty about the factor is not accounted for. A disadvantage of the BC-SMD approach is that the effect size can only be calculated for studies that include at least three cases, and that by aggregating the data across cases per study the opportunity is lost to explain variability between cases by including case characteristics. Also the approach of Joo et al. (2022) requires multiple cases per study. For a further discussion of within-case (including overlap) effect sizes and between-case effect sizes, we refer to Maggin et al. (2019) and Manolov et al. (2022).

Also when there are time trends, a multilevel effect size meta-analysis can be used: in a first step, estimates of the two coefficients that express the size of the effect (*b*_2*jk*_ and *b*_3*jk*_) and their corresponding standard errors can be obtained per case using OLS regression. Next, these effect sizes are combined across studies by means of two separate meta-analyses (Equation 8). An alternative is to perform a multivariate meta-analysis (Kalaian & Raudenbush, 1996) that accounts for the sampling covariance (also estimated in an OLS regression) between both regression coefficients, and allows for estimating the covariances between the random effects at the case and study level.


8 \[\begin{array}{ccccc}
{b_{2jk}}\,{\mathrm{ = }}\, & \left({{\beta _2}\,{\mathrm{ + }}\,{v_{2k}}\,{\mathrm{ + }}\,{u_{2jk}}} \right)\,{\mathrm{ + }}\,{r_{2jk}}\\
{b_{3jk}}\,{\mathrm{ = }}\, & ({\beta _3}\,{\mathrm{ + }}\,{v_{3k}}\,{\mathrm{ + }}\,{u_{3jk}})\,{\mathrm{ + }}\,{r_{3jk}}
\end{array}\]

To study the moderating effects of case and study characteristics, Equations 6 and 8 can be extended in a similar way as the models for raw data. Before discussing additional extensions, we look at the statistical performance of the use of basic multilevel models.

## A Statistical Evaluation of the Use of the Basic Multilevel Models

In an extensive simulation study including 648 conditions (combining different levels of number of studies, number of cases per study, number of measurement occasions, the size of the immediate effect, the size of the effect on trend, between-case variances and within-case variances), Moeyaert et al. (2014a) showed that the **three-level analysis of raw data** including time trends (Equation 3) using REML results in unbiased estimates of the immediate treatment effect and the treatment effect on the time trend. Also standard errors were appropriate in general, as were the 95% confidence interval coverage proportions. Regarding power, it was found that the number of cases per study and the number of measurement occasions are much less influential than the number of studies. When the number of studies is only 10, the power is sometimes unacceptably low, especially if the variance between studies and cases gets larger, and the interest is in the effect on the time trend. Joo and Ferron (2019) found that the approach is relatively robust for a violation of the assumption of normality for the within-case residuals: the treatment effects are estimated accurately and without bias for various levels of skewness and kurtosis, and confidence interval coverage proportions and Type I error rates were acceptable.

In a second part of their simulation study, Moeyaert et al. (2013a) found that after **standardizing the data** (by dividing the scores per case by the OLS estimate of the residual standard deviation), the effect sizes are positively biased (about +15%) for short time series of 10 measurements, but this bias disappeared for longer time series. As a result, the confidence interval coverage proportion is too small when the number of measurements is small (especially when the number of studies is large and the between-case and between-study variances are small). Otherwise, results are similar as for unstandardized data. The bias due to standardizing the data can however be removed by multiplying the standardized raw data per case by the correction factor *c*(*m*) that was proposed by Hedges (1981) to correct standardized mean differences for small-sample bias due to the standardization:


9 \[c\left(m \right)\,{\mathrm{ = }}\,\frac{{\Gamma \left({\frac{m}{2}} \right)}}{{\sqrt {\frac{m}{2}} {\mathrm{}}\Gamma \left({\frac{{m-1}}{2}} \right)}}\, \approx \,1\,-\,\frac{3}{{4m\,-\,1}},\]

with m equal to the degrees of freedom, which is equal to the number of measurements minus 1, minus the number of predictors (hence *m* is equal to *n*–2 if Equation 1 is used, or equal to *n*–4 if also a time trend and its interaction with the treatment dummy is used) (Jamshidi, et al. 2021). The standardization of the data per case therefore leads to additional complexities and possible biases (also see further). Moreover, Joo et al. (2022) found that the MSE of standardized effect estimates is larger. Therefore, we recommend using unstandardized data when data are immediately comparable across studies.

Ugille et al. (2012) found in their simulation study very similar results if **effect sizes** rather than raw data are combined, with a positive bias in effect sizes for short time series if the data are standardized, resulting in a too small coverage proportions, especially when the number of studies is large and the between-case and between-study variances small. In a follow-up study, Ugille et al. (2014) showed that also for standardized effect sizes the bias can be removed by multiplying the effect sizes using Hedges’ correction factor *c*(*m*). They also found that an equally effective, yet more complex and computing-intensive approach is to use bootstrapping (Van den Noortgate & Onghena, 2005) to remove the bias in the aggregated effect size estimates. Given that within the same study multiple cases are typically measured on the same scale, still another approach is to run a two-level analysis per study in a first step in order to get a more precise estimate of the within-case standard deviation, and use this estimate to standardize the data. Yet, Ugille et al. (2014) found that this approach does not succeed in eliminating all bias. Therefore, it is recommended to use Hedges’ bias correction factor routinely.

Regarding the **variance estimates**, Moeyaert et al. (2014a) found substantial bias in the between-study and between-case variance estimates for the immediate effect and for the effect on the time trend (up to about 50%, when the true variance is relatively small and estimated on small datasets). Also in other studies (e.g., Moeyaert, Ugille et al., 2016; Owens & Ferron, 2012; Petit-Bois et al., 2016), it was found that, even if the model was correctly specified, fewer than 30 studies may not be sufficient to avoid substantial bias and imprecise estimates of the between-study variance. When analyzing standardized data, applying Hedges’ (1981) bias correction factor when the number of measurements is small also reduces the amount of bias in variance estimates between cases and between studies (Jamshidi et al., 2021).

One choice one has to make when using the multilevel approach to SCED meta-analysis is the choice between combining the **raw data**, or first calculating the regression coefficient estimates per case and then combining them over cases. Results are very similar, although for more complex models such as models with a quadratic time trend, it is somewhat more unlikely that the analysis of raw data will converge (Declercq et al., 2022). Also for the variance components, Declercq et al. (2022) found, in general, very similar results for both approaches, although for complex models the raw data analysis might lead to even more biased variance estimates. Jamshidi et al. (2021) found that the between-case variance estimates are somewhat less biased when using effect sizes, whereas for the between-study variance, the raw data analysis results in less bias. An important advantage of using raw data instead of effect sizes, however, is the flexibility. As shown further on, raw data models can easily be adapted to account for autocorrelation, the discrete nature of the data, or various time trends. Calculated effect sizes, such as standardized mean differences, and their corresponding sampling variances may ignore these characteristics of the data, resulting in flawed meta-analytic results (Chen & Pustejovsky, 2022).

When aggregating effect sizes across cases when there are multiple effect sizes per case (e.g., *b*_2*jk*_ and *b*_3*jk*_ from Equation 8), another choice one has to make is between separate univariate meta-analyses or a single **multivariate meta-analysis**. In general, the overall treatment effect sizes and variances seem to be slightly better estimated when using a multivariate model, even if higher-level covariances between effect sizes are ignored (Jamshidi et al., 2020).

## Extending the Multilevel Models

The basic multilevel model can be extended in many ways. Here we discuss some extensions regarding the fixed part, the random part and the outcome variables.

### Extending the Fixed Part

Above, we illustrated how within cases, we can model the variation by accounting for a treatment dummy indicator, and how we can include a linear time trend. Alternative options are possible to model time trends. For instance, Mulloy (2011) and Shadish et al. (2013) proposed to use a quadratic function to model nonlinear time trends in the treatment phase. Yet, a pattern that is not uncommon in SCED research is that data show an increase in the response after the treatment is given and this increase flattens until an upper asymptote is reached. A quadratic model could fit well this pattern if the maximum value is not reached before the end of the time series, but if the study lasts longer, the quadratic model would predict that the score is going down again. A more complex model to study this type of nonlinear time trends in one study with multiple cases was studied by Hembry et al. (2015):


10 \[{Y_{ij}}\,{\mathrm{ = }}\,{\beta _0}\,{\mathrm{ + }}\,{u_{0j}}\,{\mathrm{ + }}\,(\frac{{\left[ {f\,{\mathrm{-}}\,\left({{\beta _0}\,{\mathrm{ + }}\,{u_{0j}}} \right)} \right]}}{{\left\{ {1\,{\mathrm{ + }}\,exp\left[ {-\,a(Tim{e_{ij}}\,-\,b} \right]} \right\}}}){D_{jk}}\,{\mathrm{ + }}\,{e_{ij}}\]

with *e*_*ij*_ assumed to be normally distributed with zero mean and variance \[\sigma _A^2\] for the baseline phase, and \[\sigma _B^2\] for the treatment phase. As before, *Time* is centered so that it is zero at the start of the intervention phase. *β*_0_ + *u*_0*j*_ reflects the baseline level, as well as the lower asymptote in the treatment phase. *f* refers to the upper asymptote, *b* to the inflection point of the s-shaped curve in the treatment phase, and *a* to the velocity of the nonlinear trend. *f, a* and *b* were considered by Hembry et al. (2015) as fixed, but a possible extension is to allow the parameters to vary randomly or in function of moderator variables. The authors found via simulations that these types of trajectory are relatively well recovered by this model, even when data from only 3 cases with each 10 measurements are available, although the estimation of the *b*-parameter and the residual variance in the treatment phases, \[\sigma _{\mathrm{B}}^2\], appeared to be difficult.

A drawback of the previous models that include time trends is that they require the researcher to specify a functional form for the trend, whereas in reality this form is unknown. A misspecification of the functional form can result in flawed parameter estimates and standard errors. Shadish, Zuur, and Sullivan (2014) present and illustrate the use of multilevel generalized additive models (GAMs). This flexible semiparametric approach combines parametric prediction with nonparametric smoothing techniques to make the empirical data determine whether there is a time trend and to model accurately the exact trend in the data. Finch and Finch (2018) found in their simulation study that the approach produced accurate estimates of intervention effects and kept the Type I error rate under control, even when there were as few as 15 measurements per case.

A second type of extensions are meant to account for various alternative designs. Given that a MBD is the most commonly used design in SCED research, methodological work often focused on this type of design. Shadish and Sullivan (2011) studied 809 SCED studies published in 2008 in the psychological and educational field, and found that the most commonly used designs were the MBD (54.3% of the studies), reversal designs (8.2%) and alternating treatment designs (8%). In a simple ABAB reversal design, a first ‘block’ of baseline phase and treatment phase is followed by a second block, allowing to explore whether if the treatment stops, the scores are returning to the baseline level, and if the treatment starts again the treatment effect is manifested again, enhancing the internal validity of the design. The data for one case could be modelled by extending Equation 1, by including two additional indicators, *Block*1 and *Block*2, that are equal to 1 if the score was obtained in the first or second block respectively (Van den Noortgate & Onghena, 2003a).


11 \[{Y_i}\,{\mathrm{ = }}\,({\beta _1}\,{\mathrm{ + }}\,{\beta _2}{D_i}){(Block1)_i}\,{\mathrm{ + }}\,({\beta _3}\,{\mathrm{ + }}\,{\beta _4}{D_i}){(Block2)_i}\,{\mathrm{ + }}\,{e_i}\]

The four coefficients refer to the expected baseline score and treatment effect in the first block, and the expected baseline score and treatment effect in the second block, respectively. The model can be extended in a similar way as discussed above to a two-level or three-level model to analyze data across multiple cases in a study, or across studies. Moeyaert et al. (2014b) showed how the predictor variables can be coded in alternative ways, to make sure the parameters are easily interpretable by the researcher in light of the research questions, and how the model can be extended to account for possible time trends.

In an alternating treatment design, two or more treatments are alternated rapidly over measurements (possibly following a baseline phase). One way to model data from two treatments is by using two dummy indicators for the two treatments (Van den Noortgate & Onghena, 2007):


12 \[{Y_i}\,{\mathrm{ = }}\,{\beta _0}\,{\mathrm{ + }}\,{\beta _2}{(D1)_i}\,{\mathrm{ + }}\,{\beta _3}{(D2)_i}\,{\mathrm{ + }}\,{e_i}\]

*β*_2_ and *β*_3_ can now be interpreted as the treatment effect for both treatments compared to the baseline level, but alternative coding can be used if the interest is in a direct comparison of both treatments, and the model can be extended to account for time trends (Moeyaert et al., 2014b).

Shadish and Sullivan (2011) found that a substantial part (26.9%) of the SCED studies they examined used designs that combine features of various basic designs, including 12.2% of the studies that used a mixture of a MBD and reversal design, and 9.9% of the studies using a combination of MBD and alternating treatment designs. Again, the flexibility of the regression models makes it possible to account for these mixed designs (Moeyaert, Akhmedjanova et al., 2020).

A risk in estimating and combining case-specific effects is that these case-specific effects may be biased due to external events or model misspecification (e.g., a missspecification of the time trend). In contrast, looking within a MBD study at the data across multiple cases at a given time point where some cases are still in the baseline phase, whereas for others the treatment already started, is less prone to bias. Therefore, Ferron et al. (2014) proposed a model that combines a within-case model and a between-case model, and used two non-overlapping subsets of data to estimate the parameters of both parts. Because for fitting the between-case model fewer data points are used, the power for testing the mean effect parameter from the between-case part of the model is small and parameter estimates are less precise, but estimates are unbiased and Type I error rate is under control (Ferron et al., 2014; Joo et al., 2022). This is even the case if there are event effects, which eases making causal inferences. As a consequence, estimating and testing the difference between the between-case and within-case effect estimate can be used to detect model misspecifications. Joo et al. (2022) however remark that the between-case estimator is based on the assumption of random assignment of cases, and they showed via simulation that if most problematic cases are assigned to the shortest baselines, the between-case estimator can be seriously biased.

Another extension of the fixed part that we want to present is meant to account for possible external effects in a study. Moeyaert et al. (2013b) proposed to correct for possible external effects while estimating the effects for each case by means of an OLS regression analysis per study, before combining these coefficients using a multilevel model. For a study including two cases, the following model can be used:


13 \[\begin{array}{ccccc}
{Y_{ij}}\,{\mathrm{ = }}\, & {\beta _{01}}{P_{1j}}\,{\mathrm{ + }}\,{\beta _{02}}{P_{2j}}\,{\mathrm{ + }}\,{\beta _{11}}Tim{e_{i1}}{P_{1j}}\,{\mathrm{ + }}\,{\beta _{12}}Tim{e_{i2}}{P_{2j}}\,{\mathrm{ + }}\,{\beta _{21}}{D_{i1}}{P_{1j}}\,{\mathrm{ + }}\,{\beta _{22}}{D_{i2}}{P_{2j}}\\
& {\mathrm{ + }}\,{\beta _{31}}{D_{i1}}Tim{e_{i1}}{P_{1j}}\,{\mathrm{ + }}\,{\beta _{32}}{D_{i2}}Tim{e_{i2}}{P_{2j}}\,{\mathrm{ + }}\,\mathop \sum \limits_{m = 2}^{I - 1} {\beta _{\left({m + 2} \right)}}{M_{mi}}\,{\mathrm{ + }}\,{e_{ij}}
\end{array}\]

With *P*_1_ and *P*_2_ being dummy variables for the two cases, and *M*_mi_ being an indicator for the *m*-th measurement occasion, which is equal to 1 if measurement *i* was done on this occasion (*m* = *i*), zero otherwise. The idea is that if something happens at a specific time point in a study (e.g., change of the teacher in a class), this will affect all cases from that study at that time point, and the coefficient at that time point is expected to be different from zero. The coefficients *β*_0*j*_, *β*_1*j*_, *β*_2*j*_, and *β*_3*j*_ still refer to the intercept and time trend during baseline, the immediate treatment effect and the effect on the slope for case *j*, respectively, but are now corrected for a potential external event effect. In principle, a two-level model (with random case effects) could be used, but the advantage of this model without random effects is that in this way immediately separate estimates of the coefficients are obtained for each case, and this model can be used also if only very few cases are observed in a study. In a next step, the regression coefficients can be combined using meta-analytic techniques (Equation 8). Moeyaert et al. (2013b) showed that this model is indeed able to avoid that external event effects bias the effect estimates, and results in accurate (although slightly conservative) confidence interval proportions.

A related extension was proposed by Chiu and Roberts (2018) in order to determine breakpoints in the data, these are measurement occasions at which the data pattern seems to change. Dummy variables can be constructed that are equal to zero before the potential breakpoint, and one on and after that point. By comparing the model fit (i.e., BIC) of models that differ in what dummy variables are included, one can determine whether and when there are breakpoints, and explore whether a breakpoint coincides with the intervention.

### Extensions Regarding the Outcomes

A first complexity regarding the outcome variables, is that in SCED research the outcome is often a count, a rate, or a percentage. In their review of SCED studies published in 2008, Shadish and Sullivan (2011) found that in more than 90% of the studies the outcome was count-based. These can be counts over a period of time (e.g., number of social interactions during an observation session), or counts out of a certain fixed number of trials (often expressed as a rate or proportion; e.g., the number/proportion of time intervals out of 10 intervals in a session during which there was social interaction). Yet, the models presented above assume that data are continuous and normally distributed, which does not make much sense when the expected count is small or when the expected proportion is close to zero or one. Nagler, Rindskopf, and Shadish (2008) made use of a generalized multilevel model to handle count data for MBD studies. Extending their models for multiple studies (Rindskopf, 2014; Shadish et al., 2013) leads to:


14 \[\begin{array}{*{20}{c}}
{{Y_{ijk}}\,\sim\,{\mathrm{Poisson}}({\lambda _{ijk}})}&{{Y_{ijk}}\,\sim\,{\mathrm{binomial}}({n_{ijk}},\,{\pi _{ijk}})}\\
{\log \left({{\lambda _{ijk}}} \right)\,{\mathrm{ = }}}&{\log (\frac{{{\pi _{ijk}}}}{{1\,-\,{\pi _{ijk}}}})\,{\mathrm{ = }}}\\
{\left({{\beta _0}\,{\mathrm{ + }}\,{v_{0k}}\,{\mathrm{ + }}\,{u_{0jk}}} \right)}&{\left({{\beta _0}\,{\mathrm{ + }}\,{v_{0k}}\,{\mathrm{ + }}\,{u_{0jk}}} \right)}\\
{{\mathrm{ + }}\,\left({{\beta _1}\,{\mathrm{ + }}\,{v_{1k}}\,{\mathrm{ + }}\,{u_{1jk}}} \right)Tim{e_{ijk}}}&{{\mathrm{ + }}\left({{\beta _1}\,{\mathrm{ + }}\,{v_{1k}}\,{\mathrm{ + }}\,{u_{1jk}}} \right)Tim{e_{ijk}}}\\
{{\mathrm{ + }}\,\left({{\beta _2}\,{\mathrm{ + }}\,{v_{2k}}\,{\mathrm{ + }}\,{u_{2jk}}} \right){D_{ijk}}}&{{\mathrm{ + }}\,\left({{\beta _2}\,{\mathrm{ + }}\,{v_{2k}}\,{\mathrm{ + }}\,{u_{2jk}}} \right){D_{ijk}}}\\
{{\mathrm{ + }}\,({\beta _3}\,{\mathrm{ + }}\,{v_{3k}}\,{\mathrm{ + }}\,{u_{3jk}})Tim{e_{ijk}}\,{\mathrm{*}}\,{D_{ijk}}}&{{\mathrm{ + }}({\beta _3}\,{\mathrm{ + }}\,{v_{3k}}\,{\mathrm{ + }}\,{u_{3jk}})Tim{e_{ijk}}\,{\mathrm{*}}\,{D_{ijk}}}
\end{array}\]

For the count outcome variable, a Poisson model with expected count equal to *λ*_*ijk*_ is assumed. This expected count is written as a function of time, the treatment dummy and its interaction and case and study random effects. For proportions, a binomial distribution can be used, with *n*_*ijk*_ the total number of trials at time *i*, for case *j* in study *k*, and *π*_*ijk*_ the expected rate. Note that for both the Poisson and binomial distribution, the variance is an exact function of the expected value. In reality, this relationship may not necessarily hold. To model possible overdispersion in count data (possibly caused by autocorrelation), one could make use of a quasi-Poisson distribution that includes an overdispersion parameter, or a negative binomial distribution (a Poisson-gamma mixture distribution) (Shadish et al., 2013; Li et al., 2023). Similarly, for proportion data beta-binomial models can be used. For an in-depth description and empirical illustrations of these models and further extensions (such as the use of an additional normally-distributed random residual at the first level of the generalized model to deal with extra noise), we refer to Li et al. (2023; 2024). Li et al. (2023) also show using simulation that ignoring overdispersion may lead to equally efficient effect estimates, but flawed inferences. Declercq et al. (2019) found that the basic multilevel model for normal outcome variables still may perform reasonably well when in reality the outcome variable is a discrete count; whereas the model fit and power was somewhat worse compared to when the correct model (Equation 14) is used, the Type I error rate was found to be even somewhat better under control. Yet, Li et al. (2023) warn that we have to be prudent when treating the outcome as normally distributed, especially when the expected count is small and/or if the variance of the random effects is large, because in these cases the distribution of counts deviates clearly from a normal distribution. Similarly, for proportions, we need to be especially prudent when the expected number of occurrences or non-occurrences is 5 or smaller.

Another complexity is that sometimes studies evaluate the effect of a treatment using multiple outcomes. One way to model the dependence of effects, is to define an additional level, this is a level for the outcomes (Chiu & Roberts, 2018; Van den Noortgate et al., 2013, 2015). For the meta-analysis of SCED studies, this would mean that we could use a four-level model: For each study (Level 4), we may have multiple cases (Level 3), for which multiple outcomes were measured (Level 2) using repeated measurements (Level 1). Note that by using this model, we consider the outcome variables for each case as a random selection of outcomes. If the outcomes are the same for all cases within a study, the structure is rather a cross-classified structure (Fernandez-Castilla et al., 2019): Within studies (Level 3), we have a cross-classification of outcomes and cases (Level 2), and within these combination, we have repeated measurements (Level 1). If only a few (types of) outcome variables are used across studies, one could include dummy outcome indicator variables as well as interaction terms between these dummy variables and the dummy treatment indicator in the four-level model, in order to get a better insight in the differences between outcomes in the baseline level and effects of the intervention, respectively. By dropping the main effect of the treatment indicator while including its interaction with each dummy outcome indicator variable, one gets an estimate and test of the treatment effect on each outcome variable. By keeping the treatment indicator effect, but not including the interaction term for one (reference) outcome variable, one gets an estimate and test of the contrast of the treatment effects for the various outcome variables compared to the reference outcome variable. To account for possible heterogeneity, separate within-case variances can be estimated for the different outcome variables, as well as covariances between pairs of outcomes. At the higher levels, the additional regression coefficients can be defined as random with a covariance matrix that has to be estimated, resulting in a full multivariate model. Advantages of this multivariate approach are that it also gives insight in the correlation between the outcomes (and the treatment effects for the different outcomes) at the within-case, between-case, and study level, and that it can handle heterogeneity between outcomes in the variance at the different levels (Baek et al., 2022). A drawback is that the model gets much more complex, especially if all regression weights are allowed to vary randomly with an unconstrained covariance matrix. One solution is to simplify the model. For instance, Baek et al. (2022) proposed to make the outcome contrasts fixed, which means assuming that these contrasts do not vary within cases, across cases, and across studies. As an alternative approach when sufficient data are available for each outcome, one can consider performing a separate multilevel analysis for each outcome.

### Extending the Random Part

In Equations 2 and 3, we assumed that the within case residuals are identically and independently normally distributed. This is a strong assumption in time series. Especially if the measurements are following closely in time, it is likely that subsequent measurements are affected by similar influences and therefore are dependent. Based on their review of SCED studies, Shadish and Sullivan (2011) estimated that the average autocorrelation is about .20. Ignoring a positive autocorrelation is expected to lead to underestimated standard errors and therefore inflated Type I error rates and too small confidence interval proportions (Greenwood & Matyas, 1990), and more biased variance estimates, also in two-level SCED analyses (Ferron et al., 2009). To account for autocorrelation, one could for instance assume that the residuals are the result of a first-order autoregressive process, AR(1), that is, it is assumed that the correlation between two adjacent scores from a given case is equal to ρ, the correlation between two scores that are two time units apart from each other is ρ^2^, the correlation between scores with three time units in between is ρ^3^, and so on. Petit-Bois et al. (2016) found in their simulation study that multilevel SCED analyses allowing for first-order autoregressive residuals yielded appropriate fixed effect estimates and confidence intervals, regardless of whether data were simulated with autoregressive residuals (i.e., the analysis model was correctly specified), independent residuals (i.e., the analysis model was overspecified), or residuals generated using a moving average model (i.e., the analysis model was misspecified). Baek and Ferron (2013) went a step further and used Bayesian estimation for a model with an AR(1) structure for the level-1 residuals, but with an autocorrelation and variance that differed across cases from a MBD study. In their simulation study, Baek and Ferron (2020a) found that the fixed effect estimates and inferences are hardly impacted by incorrectly assuming homogeneity in the level-1 covariance matrix across cases (unless in extreme heterogeneity conditions), but variance estimation was more affected. Especially the estimates of the level-1 autocorrelation and variance were found to be biased and confidence interval coverage were flawed when incorrectly assuming a common covariance matrix across cases. Chen and Pustejovsky (2022), however, found that ignoring autocorrelation may also lead to strongly biased fixed effect estimates and inappropriate confidence interval coverage. A possible explanation of these seemingly disparate results is that whereas Petit-Bois et al. (2016) and Baek and Ferron (2020a) studied the analysis of unstandardized data generated from normal distributions, Chen and Pustejovsky (2022) studied mean differences that were standardized per case, based on count data generated using shifted Poisson distributions.

Another way in which the assumption of identically and independently normally distributed within-case residuals might be violated is in case the treatment affects the variability in scores. In this situation of heterogeneity, data can be further analyzed using a model including separate variances for both conditions (Van den Noortgate & Onghena, 2003a). If data should be standardized, one can use the within-case standard deviation for the baseline condition only (Bunuan et al., 2013; Joo et al., 2019).

Also at the second and third level (the case- and study-level respectively), we can model alternative (co)variance structures. The simplest structure is assuming that at each level the residuals (the *u*’s at the case level, and the *v*’s at the study level) are independent. A more complex structure is to use an unrestricted covariance matrix, that is, to estimate the covariance between the random effects as well. Ignoring an existing covariance however does not largely affect the quality of the estimates or statistical inferences for the treatment effects (Moeyaert, Ugille et al., 2016).

A final extension of the random part is the inclusion of additional random effects to model variation across other kinds of units. We already mentioned the use of random effects to model the variation between outcomes, resulting in a four-level or a cross-classified model, depending on whether or not we can consider the sets of outcomes measured within studies as study-specific. Debodinance et al. (2017) included an intermediate level (between the level of measurement occasions and the level of cases) to take into account variation between multiple time-series that were reported for the same case. Another scenario is where studies are nested in research groups (i.e., some research groups did multiple studies). In this case, the resulting dependence in study results (studies from the same group may be set up in a similar way and hence yield more similar results) can be accounted for by defining a new upper level.

## Estimation and Significance Testing

For estimating the parameters of multilevel models, often maximum likelihood methods are used. For synthesizing SCED data, restricted maximum likelihood (REML) is recommended given the small number of units typically available (Owens & Ferron, 2012). For testing the fixed parameters, usually the ratio of the maximum likelihood estimate over the estimated standard error is compared to a normal or a *t*-distribution. Various approaches exist to approximate the degrees of freedom of the t-distribution. In the context of multilevel modelling of MBD data, Ferron et al. (2009) compared five approaches that are implemented in SAS PROC MIXED: the containment, the residual, the between-within, the Satterthwaite, and the Kenward-Roger method. The Kenward-Roger method also implies an inflation of the standard errors of the fixed effects, in order to account for the uncertainty in the variance component estimates. They found that the Satterthwaite and Kenward-Roger methods, that were particularly developed to improve multilevel modelling results for small sample sizes, yield appropriate confidence intervals for the regression coefficients. With an increasing number of cases, the coverage proportions of the other methods improved, but remained biased.

An alternative is the use of Bayesian methods. Moeyaert et al. (2017), Baek et al. (2020) and Joo and Ferron (2019) compared the parameter recovery when using the Bayesian framework (with weakly or noninformative priors, respectively) or when using maximum likelihood for estimating the basic two-level model parameters for one MBD study. Although in theory, an advantage of the Bayesian approach is that it takes into account the uncertainty of estimating both the fixed and the variance parameters (Gelman, Carlin, Stern, & Rubin, 2004), both approaches seem to yield very similar results: For the fixed parameters, no bias was observed and the Type I error rate was under control for both approaches; for the variance components, Bayesian methods did not succeed in reducing the bias in estimating the variance at the highest level that is especially observed when data from only a small number of cases (i.e., 3) are available. Joo and Ferron (2019) even found that the Bayesian approach may lead to a slight undercoverage of the confidence intervals when the number of observations increases. Yet, an important strength of the Bayesian framework is that it is applicable for models of high complexity. As an example, Hembry et al. (2015) resorted to Bayesian methods to fit the model with nonlinear time trends (Equation 10). Baek, Petit-Bois, and Ferron (2013) found that while the REML failed to converge when used for a model with a separate level-1 variance parameter per case, the model could be estimated using a Bayesian analysis. Moreover, Bayesian analysis may lead to more accurate results if only a small or unequal number of measurements is available per case (Joo & Ferron, 2019; Rindskopf, 2014; Shadish et al., 2013). Another advantage of the Bayesian approach is that it can be used to bring in prior knowledge about the model parameters, which has been shown to be able to improve the precision and bias of the variance components especially if the number of cases is very small (Moeyaert et al., 2017b), but more research has to be done on the use of different prior distributions for SCED meta-analysis.

An alternative for testing the fixed effects is using the Robust Variance Estimation (RVE) procedure proposed by Hedges, Tipton, and Johnson (2010) to obtain sandwich estimates of the standard errors (Huber, 1967; White, 1982). The advantage of this approach is that it is expected to yield an asymptotically consistent covariance matrix of the fixed effects estimates, even if the random part of the model has been misspecified. Specific for SCED meta-analyses, simulation research nevertheless showed that the use of RVE does not always lead to better standard error estimates (Jamshidi et al., 2020). Moreover, Chen and Pustejovsky (2022) found that the use of RVE for the meta-analysis of standardized mean differences cannot compensate for a misspecification of the sampling variances due to ignoring the discrete nature of the data and possible autocorrelation: Effect size estimates may be strongly biased and confidence interval coverage proportions may be far off. We conclude that although RVE may improve statistical inferences, it is not a panacea for all kinds of misspecifications.

Another way for testing the fixed effects of the multilevel models for randomized SCEDs (e.g., in which the start point of the intervention is randomly determined) is the use of a randomization test wrapper (Michiels et al., 2020). This approach is based on the idea that if there would be no treatment effect, an alternative choice of the start point would not have resulted in another time series. In the randomization test wrapper, the multilevel model is re-estimated for a large number of alternative randomizations for the different cases. For the overall treatment effect (or another parameter of interest), we therefore obtain a distribution of estimates that can be used as the reference distribution to make inferences (instead of approximating the null distribution by a normal or *t*-distribution). By inverting the randomization test, it is possible to construct confidence intervals for the treatment effect (Michiels et al., 2017). An advantage of the randomization test approach is that it does not make distributional assumptions about the residuals and it does not require random sampling. Michiels et al. (2020) showed that when the residuals are not identically and independently normally distributed, this approach better keeps the Type I error rate under control and at the same time may result in higher power when testing the mean treatment effect of a multiple baseline design. Moreover, a randomization test can be used for exploring various data features that are often considered in SCED research, including the effect on level, on trend, and on variability, as well as overlap and immediacy (Tanious et al., 2019).

## Discussion

In this paper, we gave an overview of the multilevel regression approach to meta-analyze SCED data and of its performance. The use of multilevel models is an elegant way to statistically account for the inherently nested structure of SCED data (with measurements nested in cases and cases nested in studies). It also allows going further than estimating effects for individual cases, by estimating and testing the overall effect, the variation between cases and studies, and by exploring factors that affect the size of the effect. We also showed how the flexibility of the multilevel approach can be exploited to account for many different complexities. We believe that this overview comes at a good time, because of the explosion of methodological and statistical research that has been done in the last decade on this new technique, resulting in many papers scattered across journals. We believe that this overview will also make researchers aware of the opportunities offered by the multilevel approach of SCED meta-analyses and give researchers guidance in tailoring the models to the specificities of their data and research questions. In addition, we hope it can contribute to an increased attention for (the meta-analysis of) SCED studies in statistical training of students in the behavioral sciences, which nowadays still primarily focusses on group studies.

The scope of the paper has however some limitations. First, the multilevel approach is only one approach to handle SCED data. We did not discuss alternative approaches to (meta-)analyze SCED data, including the combination of *p*-values from randomization tests executed per case to test the existence of a treatment effect (Heyvaert et al., 2014; Onghena et al., 2018). For an overview and discussion of various methods, we refer to Manolov and Moeyaert (2017). Second, we did not include real-data examples to illustrate the multilevel approach. This is because the basic multilevel approach has been illustrated before in multiple papers (e.g., Van den Noortgate & Onghena, 2008; Moeyaert, Ferron et al., 2014). For real data illustrations of the different (extensions of) multilevel models and the different approaches discussed above, we refer to the respective papers that were cited in the text. Third, the paper is not meant as a practical tutorial for doing a multilevel meta-analysis of SCED studies, again because this can be found in previous papers (see, e.g., Becraft et al., 2020; Moeyaert et al., 2020). Various general or specific multilevel modeling software programs can be used to do the analyses, such as HLM, MLwiN, SAS, SPSS, R, Mplus, Stata, and OpenBugs. Baek et al. (2022) discuss these options and describe the syntax for doing multilevel analyses of SCED data using SAS PROC MIXED (also see Moeyaert et al., 2013a), using the lme4 package of R, and using OpenBUGS (for Bayesian analysis; also see Baek & Ferron, 2020b). Pustejovsky et al. (2023) developed an R-package, scdhlm, and a web-based calculator for estimating multilevel model parameters and design-comparable effect sizes for SCED designs with multiple cases, such as multiple baseline designs, multiple probe designs or replicated treatment reversal designs. A user-friendly R-based point-and-click tool that can help to perform basic multilevel analyses of raw SCED data is multiSCED (Declercq et al., 2020), which is a free Shiny app that comes with a step-by-step tutorial based on a built-in example dataset. A strength of the tool is that it can also be used to supplement visual inspection of the data, by superimposing in the time series graphs the estimated trajectories based on a one-level, two-level, or three-level model, as proposed by Baek et al. (2016). In this way, applied researchers are helped to interpret the results of the multilevel analyses, detect outlying scores, cases or studies, and detect model misspecifications. Moreover, the tool automatically shows the R code that is generated while specifying the model using the menus, giving researchers a first base for exploring more advanced analyses in R.

We also would like to make some reflections on multilevel meta-analyses of SCED studies. First, multilevel models are extremely flexible. The models presented above can be further extended to loosen some assumptions (for instance, in the nonlinear model presented above, Equation 10, one could make the upper asymptote, the inflection point, and the velocity parameter random over cases). Moreover, several extensions can be combined (e.g., nonlinear time trends, autocorrelation, and the effect of external events). At the other side of the coin, this flexibility results in the challenging task for researchers to choose a good model or approach. There is indeed no uniformly best model, so researchers should specify a model that fits well in their situation. Still we try to give some general recommendations. First, we recommend standardizing the data only if they are not measured on the same scale, and when data are standardized, using Hedges’ bias correction factor. A second recommendation is to tailor the model to the kind of data available. If data are clearly discrete (e.g., counts, especially when the expected count is small), the use of a generalized multilevel model is a good idea. We also want to advocate a thorough data exploration before choosing a model, for instance by means of the time series graphs that are typically reported in primary studies. This can help to identify for instance outlying values, skewness (which may be solved by means of a data transformation), autocorrelation and time trends. Also after doing an analysis, a visual inspection of the residuals (e.g., using a histogram) or plotting the multilevel regression curves in the time series graphs can help to assess model misspecification. Researchers should also reflect on dependencies present in the data. For instance, if there are multiple outcomes per study or if studies are nested in research teams, an additional level can be included in the model. A third recommendation is to account for theory. If from a theoretical point of view, you might expect that after intervention, problem behavior will gradually decrease, it is a good idea to account for such a possible time trend. Fourth, also the research interests or questions can guide model selection: if one is interested in the difference between boys and girls in the treatment effect, one should include this moderator in the model. A fifth recommendation is to try to avoid overly complex models. While a more complex model might be more realistic, a drawback is that the more complex the model is, the harder it may become to obtain stable parameter estimates. In some studies, it was found that if the dataset is relatively small, a misspecified but simpler model can perform equally good or even better than the correct model. This can be the case when using a multilevel model assuming normal residuals for count data, rather than using a more complex generalized multilevel model with a Poisson distribution (Declercq et al., 2019). More research is required on the performance of even more complex models, given that SCED meta-analytic datasets are often characterized by a modest number of studies, cases per study, and measurements per case. Regarding estimation, we do not see convincing evidence for the superiority of Bayesian or REML estimation, although the Bayesian framework can give even more flexibility in model specification. To account for a possible misspecification of the model, we believe the use of robust variance estimation can be valuable (although more research is needed). As a final recommendation, we promote sensitivity analyses. If the researcher doubts between two competing models (e.g., with a linear vs. nonlinear time trend), we propose to fit both models. Besides looking at model fit indices, comparing the results gives insight into how sensitive the conclusions are to the model choices made. When the conclusions remain the same, this gives more confidence in these conclusions, whereas if they change, uncertainty increases. Of course, it is important that the researcher is transparent about what models were fitted and about the (dis)agreement in the results. Sensitivity analyses can also help to assess the sensitivity of the conclusions to other decisions, like the in- or exclusion of outliers or the estimation procedure used.

A second reflection is that the multilevel approach assumes that the units at each level can be considered as a random sample from a (normally distributed) population. In group comparison studies, study participants are often not randomly drawn, but in SCED studies, the assumption of random sampling may be even more questionable. A case may be studied exactly because the researcher is interested in this specific case, or because the researcher thinks the treatment may work for this case. A SCED meta-analyst therefore should carefully reflect on the question to what population results may be generalizable. Related to the assumption of random sampling, there may also be an issue of reporting and publication bias, this is the tendency to report or publish especially striking findings, a problem that may again be more pronounced in SCED research (Shadish et al., 2016). More research is needed to shed light on these issues in SCED research and on approaches to deal with these.

Third, an issue that requires more attention is the methodological rigor of SCED meta-analyses. Jamshidi, Heyvaert et al. (2018) used an adapted version of the AMSTAR-R to evaluate the 178 meta-analyses they found that were published between 1985 and 2015, and found that a large majority of these studies did not even reach the midpoint of the scale (22 on a scale from 0 to 44). Although they found that the methodological quality improved over time, it is clear that further improvement is required. A difficulty is that various guidelines, checklists, and quality appraisal tools are available for primary SCED studies (e.g., Horner et al., 2005; Reichow et al., 2018, Tate et al., 2013, 2016; Wendt & Miller, 2012; What Works Clearinghouse, 2020) and for meta-analyses or systematic reviews of group studies (e.g., Critical Appraisal Skills Programme, 2018; Moher et al., 2009), but guidelines or checklist for SCED meta-analysis are largely lacking (Jamshidi, Declercq et al., 2018).

Fourth, in various studies, it was found that in a wide variety of situations the fixed effects are relatively well recovered, and Type I error rates and confidence interval coverage is appropriate. This is especially true when the model is correctly specified, but inferences seem to be relatively robust to violations of assumptions (e.g., Baek & Ferron, 2020a; Jamshidi, Declercq et al., 2020; Moeyaert, Ugille, et al., 2016). Nevertheless, although the inferences may be valid, we have to keep in mind that the statistical power may be low when working with small datasets. Whereas the large effect sizes often encountered in SCED research mitigate the issue for testing the mean effect, the power for testing moderator effects may be problematic. For instance, Moeyaert et al. (2022) showed that if a two-level analysis is used to combine data from multiple cases from one study, a minimum of seven cases is recommended to test the moderating effect of a case characteristic with sufficient power (at least .80), and this number increases when more moderator variables are included in the model. Similarly, in a SCED meta-analysis the number of studies should be large enough to test the moderating effect of study characteristics. For the (co)variance components, findings on the validity of inferences are less promising. Even when the error structure is correctly specified, there can be substantial bias in the (co)variance parameter estimates when estimates are based on the small datasets that are often encountered in SCED meta-analysis (e.g., Baek et al., 2020; Hembry et al., 2015; Jamshidi et al., 2020; Moeyaert et al., 2013b; Owens & Ferron, 2012; Petit-Bois, et al., 2016; Ugille et al., 2012), and this bias is even more substantial when the variance is close to its theoretical boundary of zero and when the error structure is misspecified (Baek & Ferron, 2020a; Joo et al., 2019: Moeyaert, Manolov et al., 2020). The amount of bias that was observed seems to vary across the simulation studies, and may be due to differences between studies in whether two- or three-level data were simulated, whether data were standardized, whether Hedges’ correction factor was used, whether raw or effect size data were used, what sampling variance formula was used for standardized effect sizes, whether the model was correctly specified, whether negative variance estimates were constrained to zero, and finally in the model parameters chosen. Future research could further study systematically the factors that determine the size of bias, extending the work of Li et al. (2022). In addition, given that Moeyaert et al. (2017) found that a Bayesian approach does not solve the issue of bias unless informative priors are used, future research could examine alternative methods for estimating and testing the model parameters, such as bootstrapping (Ugille et al., 2014).

Finally, we recognize that the complexity of the multilevel approach, especially when using a more extended model, may make this approach less attractive. We therefore applaud collaboration between applied single-case researchers and quantitative methodologists and statisticians as propagated by Shadish (2014). Tutorials, user-friendly software, and didactic papers reporting on real data meta-analyses may further help researchers to adopt the existing meta-analytics techniques. We hope that also this paper can stimulate researchers to cross the threshold, and to contribute to the impact SCED studies may have on evidence-based practice.
